# Lectins from Mycelia of Basidiomycetes

**DOI:** 10.3390/ijms18071334

**Published:** 2017-06-22

**Authors:** Valentina E. Nikitina, Ekaterina A. Loshchinina, Elena P. Vetchinkina

**Affiliations:** Laboratory of Microbiology, Institute of Biochemistry and Physiology of Plants and Microorganisms, Russian Academy of Sciences, 13 Prospekt Entuziastov, Saratov 410049, Russia; nikitina_v@ibppm.ru (V.E.N.); elenavetrus@yandex.ru (E.P.V.)

**Keywords:** macrobasidiomycetes, mycelial lectins, hemagglutination, fungal morphogenesis, properties and physiological functions of lectins

## Abstract

Lectins are proteins of a nonimmunoglobulin nature that are capable of specific recognition of and reversible binding to the carbohydrate moieties of complex carbohydrates, without altering the covalent structure of any of the recognized glycosyl ligands. They have a broad range of biological activities important for the functioning of the cell and the whole organism and, owing to the high specificity of reversible binding to carbohydrates, are valuable tools used widely in biology and medicine. Lectins can be produced by many living organisms, including basidiomycetes. Whereas lectins from the fruit bodies of basidiomycetes have been studied sufficiently well, mycelial lectins remain relatively unexplored. Here, we review and comparatively analyze what is currently known about lectins isolated from the vegetative mycelium of macrobasidiomycetes, including their localization, properties, and carbohydrate specificities. Particular attention is given to the physiological role of mycelial lectins in fungal growth and development.

## 1. Introduction

Lectins are carbohydrate-binding proteins of a nonimmunoglobulin nature that can specifically recognize carbohydrates (glycosylic groups) and reversibly bind to them without modifying their covalent structure [1]. The formation of a lectin–carbohydrate complex is based on complementarity—the spatial correspondence of the lectin and carbohydrate molecules.

Because lectins are uniquely able to specifically bind glycoconjugates and have a wide range of biological activities in vivo and in vitro, they are used widely as valuable biological tools (test reagents and diagnostic agents) in biology and medicine [2,3]. They are important for biological recognition and are involved in intra- and intercellular interactions. In view of the constantly expanding sphere of application of lectins, the demand for these biologically active substances is growing steadily. Lectins are widespread in living nature, and almost all organisms, including fungi, can produce them [3].

Whereas the study of plant, animal, and bacterial lectins has been active and multifaceted, little work has been done on lectins from higher fungi and a limited set of problems has been tackled. Analysis of the available literature shows that studies of basidiomycete lectins have been reported, mostly on the isolation of lectins from fruit bodies—short-term structures with a strictly determined function. Much less work has addressed basidiomycete lectins at the dikaryotic mycelium stage, which is the principal stage of growth of the fungus as a biological organism. Very few reports have investigated lectins from basidiomycetes in connection with the physiological aspects of the fungal life cycle.

A variety of fungi, including members of the genera *Armillaria*, *Agrocybe*, *Cerrena*, *Coprinus*, *Fomes*, *Funalia*, *Ganoderma*, *Grifola*, *Gymnopilus*, *Kuehneromyces*, *Lactarius*, *Lentinus*, *Laetipous*, *Panafolus*, *Pholiota*, *Pleurotus*, *Pseudotremella*, *Punctularia*, *Pycnoporus*, *Schizophyllum*, *Termitomyces*, *Trametes*, *Tricholoma*, and *Volvariella*, can synthesize mycelial lectins. Most of these are intracellular [4,5,6,7,8,9,10], while others are associated with the surface of mycelial hyphae [11,12,13]. Some lectins are extracellular and are secreted into the growth medium during the fungal life cycle [4,14,15,16]. It has been established that lectin activity depends on a fungus’ species, strain, age, growth stage, and morphological structure, as well as on the cultivation method and growth medium used. The lectins present in the fungal mycelium may be identical to those present in the fruit body or may have completely different properties and perform different functions.

This article reviews and analyzes the available literature on lectins isolated from the vegetative mycelium of macrobasidiomycetes, with emphasis on the proposed lectin functions in the host organism. Biomedical aspects of lectin application are beyond our present scope and have been discussed at length by previous reviewers [17,18,19,20,21,22,23].

## 2. Intracellular Lectins from Mycelia of Basidiomycetes

Although the first hemagglutinins of basidiomycetes were discovered in their fruit bodies more than a hundred years ago in toxicity studies [24], research on basidiomycete mycelial lectins began to appear only in the second half of the twentieth century and is still relatively rare. At present, the best studied basidiomycete mycelial lectins have intracellular localization. One of the most important functions of basidiomycete lectins is their involvement in fungal growth and morphogenesis, in fruit body formation, in the formation of specialized morphological structures (such as those used by pathogenic fungi for invasion), and in interaction with higher plants during mycorrhiza formation (in mycorrhizal fungi) [18,19]. For this reason, a number of studies have compared lectins from fruit bodies with mycelia and other morphological structures.

The most common and convenient test system for the detection and determination of lectin activity is the hemagglutination assay. It is a simple and easy method to obtain semiquantitative data on the sugar binding and specificity of a lectin [25]. Lectins agglutinate erythrocytes through reversible binding of sugars found on the cell surface [22]. The hemagglutination assay is done in a series of lectin dilutions by using native or protease-treated erythrocytes of animal or human origin. For isolation and purification of lectins, conventional methods for purifying proteins are used, such as chromatography on various resins (affinity, ion-exchange, high-performance liquid (HPLC), and fast protein liquid chromatography) [20].

Banerjee et al. [26] examined seven mushroom species (*Volvariella volvacea*, *Termitomyces clypeatus*, *Panafolus papillionaceus*, *Gymnopilus chrysimyces*, *Lentinus squarrosulus*, *Coprinus lagopus*, and *Coprinus atramentarius*) and found that their mycelial extracts agglutinated erythrocytes of several animals. Most of the mycelia possessed strong hemagglutinins active against various erythrocytes. Sheep erythrocytes were the most sensitive, whereas goat and human erythrocytes were highly resistant to the hemagglutinins. Mycelial extracts from *T. clypeatus* and *L. squarrosulus* were most active against sheep erythrocytes. The presence of more than one hemagglutinating factor in the mushroom mycelia was observed. Extracts from *P. papillionaceus* and *G. chrysimyces* had appreciable activity against rat erythrocytes, whereas *L. squarrosulus* was active against guinea pig and mouse erythrocytes. Agglutination was partly inhibited nonspecifically by high concentrations of glucose, galactose, mannose, fucose, and rhamnose.

In studies of the relationships between lectins present in fruit bodies and mycelia, both Ticha et al., with *Agrocybe aegerita*, and Musilek et al., with *Flammulina velutipes*, *Kuehneromyces mutabilis*, and *Pholiota squarrosa*, found marked differences in the presence of lectins and in their specificity [27]. Only *K. mutabilis* mycelial lectins had properties similar to those of the lectins from wild fruit bodies. However, when *A. aegerita* mycelial cultures had matured to the phase of fruit body formation, their lectin composition was comparable to that of the wild material.

Sometimes lectins were found in the fruit bodies of basidiomycetes but not in their vegetative mycelium. For example, a homogenous lectin was purified from the fruit bodies of *Flammulina velutipes*, but immunological assays showed that it was absent from vegetatively growing mycelia [28]. Lectin activity in the basidiomycete *Pleurotus cornucopiae* was studied at different morphological stages (vegetatively growing mycelium, primordium, and immature and mature fruit body) by using human type A erythrocytes [29]. Hemagglutinating activity was not found in the vegetative mycelium, but it started to appear in the primordium stage and continued to increase during fruit body formation. A protein cross-reactive with an antilectin serum also appeared after a primordium had formed.

A protein, LZ-8, with mitogenic activity in vitro and immunomodulating activity in vivo, was isolated and purified by gel filtration and ion-exchange chromatography from a mycelial extract of the medicinal fungus *Ganoderma lucidum* [30]. LZ-8 was capable of hemagglutinating sheep erythrocytes, but no hemagglutination was observed toward human erythrocytes of any type. However, hemagglutination was not inhibited by the mono- and dimeric sugars tested by the authors, permitting the conclusion that LZ-8 is not a lectin per se [31]. Later, the lectin GLL-M (*Ganoderma lucidum* mycelial lectin) was isolated from *G. lucidum* by affinity chromatography [4]. It was isolated from combined broth and mycelial extracts, because both culture broth and crude saline extracts showed hemagglutinating activity and because preliminary experiments had indicated that the broth lectin was identical to the one from the mycelial extract. GLL-M was a monomer in its native form with a molecular mass of 18 kDa. The crude lectin fraction had no remarkable binding specificity to any simple sugar and was specific to asialo-bovine submaxillary mucin, asialofetuin, and fetuin in a hemagglutination-inhibition assay. Another lectin, GLL-F (*Ganoderma lucidum* fruit body lectin), was purified from the fruit bodies of the same fungus; and the two proteins, GLL-M and GLL-F, were compared and found to differ on the basis of their molecular size and sugar-binding properties.

A lectin, LDL (*Lactarius deliciosus* lectin), with a molecular mass of about 37 kDa was isolated from carpophores of the edible mushroom *Lactarius deliciosus* [32]. It agglutinated erythrocytes of groups A, B, and O at comparable intensities, and its hemagglutinating activity was inhibited only by *N*-acetyl-d-galactosamine and related sugars. The extract obtained from the submerged mycelium of *L. deliciosus* was also weakly but clearly agglutinating, but the lectin concentration in the mycelium was very low (at least 1000-fold lower than in the carpophores). The occurrence of lectin activity in the mycelium obtained in vitro raises the question about its role in the recognition process that takes place during mycorrhization.

Two lectins, TML-1 (*Tricholoma mongolicum* lectin 1) and TML-2 (*Tricholoma mongolicum* lectin 2), were isolated from the submerged mycelium of the edible mushroom *Tricholoma mongolicum* by ion-exchange chromatography and gel filtration [33]. They were dimers with molecular masses of about 37 kDa. The hemagglutinating activities of TML-1 and TML-2 were sensitive to lactose inhibition. TML-1 differed from TML-2 in the content of proline and tyrosine residues. Both were nonglycoproteins and had hydroxyproline residues. Later, another study ascertained the existence and characteristics of a lectin in the fruit bodies of the same species, *T. mongolicum* [34]. Unlike the mycelial lectins, the fruit body lectin was presumably a glycoprotein.

A novel lectin, VVL (*Volvariella volvacea* lectin), was purified from the cultured mycelia and fruit bodies of the edible mushroom *Volvariella volvacea* [5]. The lectins isolated from the fruit bodies and cultured mycelia were found to be identical by comparing their chromatographic behavior, N-terminal amino acid sequences, molecular weights, and biological activities. As shown by gel filtration and sodium dodecyl sulfate–polyacrylamide gel electrophoresis (SDS–PAGE), VVL was a homodimeric protein of 32 kDa. It had no carbohydrate moiety, and its hemagglutinating activity was inhibited by thyroglobulin, but not by simple carbohydrates such as monomeric or dimeric sugars. The immunomodulating activity of VVL was demonstrated by its being potently stimulatory to murine splenic lymphocytes. VVL also markedly enhanced the transcriptional expression of interleukin-2 and interferon-γ by reverse transcriptase–polymerase chain reaction (PCR). As revealed by its N-terminal amino acid sequence, VVL had a molecular structure distinct from other immunomodulatory proteins reported previously in the same fungus.

Two galectins, CGL1 (*Coprinus cinereus* lectin 1) and CGL2 (*Coprinus cinereus* lectin 2), isolated from the basidiomycete *Coprinus cinereus* (*Coprinopsis cinerea*) were shown to be differentially regulated during fruit body formation [35]. CGL2 expression was initiated early in fruit body development (hyphal knot formation) and was maintained until the fruit body matured. By contrast, CGL1 was specifically expressed in primordia and mature fruit bodies. It was tested whether these nematotoxic galectins were induced upon fungivory [36]. The induction of CGL1- and CGL2-encoding genes was examined by challenging the vegetative mycelia of monokaryotic and dikaryotic strains of *C. cinerea* with the fungivorous nematode *Aphelenchus avenae*. The expression of these genes in *C. cinerea* was analyzed at the protein level by immunoblotting with specific antibodies and was validated quantitatively at the transcript level by real-time PCR. At the protein level, in both a monokaryotic and a dikaryotic strain, bands corresponding to CGL1/2 were observed in mycelia challenged with *A. avenae*. This expression was specific for the mycelium exposed to predation, and no induction was observed on exposure to heat-killed *A. avenae*, or to live and killed cells of the bacterivorous nematode *Caenorhabditis elegans*, or on physical damage to the mycelium and young fruit bodies. The expression of both genes was, depending on the strain and medium used, between 3- and 9-fold higher than that observed in the nonchallenged mycelium, but much lower than that in fruit bodies. The induction of CGL1/2 in the monokaryotic *C. cinerea* strain suggests that the induction of the galectins by fungivory is independent of sexual development, as this strain cannot form fruit bodies. Neither *cgl1* nor *cgl2* was induced after the mycelium of *C. cinerea* was challenged with bacteria, other fungi, and cell-wall-degrading enzymes. However, it was reported that the expression of CGL1 and CGL2 in the vegetative mycelium of *C. cinerea*, even in the absence of primary hyphal knots or any other developmental structures, was induced by nutrient limitation [37]. These results indicate that fungi can regulate lectin synthesis in response to specific environmental signals, including predation [36].

A lectin named AAL (*Agrocybe aegerita* lectin), consisting of two subunits of 15.8 kDa each, was purified from the fruit bodies of the edible mushroom *Agrocybe aegerita* and was characterized [38]. The synthesis of AAL was not limited to fruit bodies, but its concentration in mycelia was much lower. AAL agglutinated erythrocytes of 12 different animals and human erythrocytes (A, B, and O type), regardless of the blood type or animal species. A hemagglutination inhibition assay showed that bovine submaxillary mucin, glycophorin A, and hog gastric mucin were strongly inhibitory, and κ-casein induced inhibition at a higher concentration. AAL promoted the differentiation of fruit body primordia from the mycelia of *Agrocybe aegerita* and *Auricularia polytricha*. The formation of fruit bodies was dose dependently accelerated by AAL dripped on the mycelium surface, providing evidence for an important role of fungal lectins in fruit body development.

Vetchinkina et al. [6,39] examined the intracellular lectins of the xylotrophic basidiomycete *Lentinus edodes*. At different stages of fungal morphogenesis, the intracellular lectins differed in their molecular mass, subunit composition, activity, and carbohydrate specificity [6]. The lectin activity of *L. еdodes* was studied during the entire life cycle of the fungus at the following morphogenetic stages, typical of this species: nonpigmented mycelium, pigmented mycelium, brown mycelial film, primordium, and fruit body. Native and trypsin-treated rabbit, sheep, cow, and horse erythrocytes were used, as were erythrocytes of the four human blood types. Trypsinized rabbit erythrocytes and, to a lesser degree, human erythrocytes were the most sensitive test objects for the *L. edodes* lectins. By contrast, the lectins almost did not react with cow, sheep, and horse erythrocytes. Hemagglutinating activity was found in extracts at all morphogenetic stages, but the titers were different. Hemagglutinating activity at the nonpigmented mycelium stage was high but increased with pigmenting and peaked at the brown mycelial film stage (65 times higher than in the nonpigmented mycelium and more than 500 times higher than in the basidiocarps). At the primordium and fruit body stages, the hemagglutinating activity decreased substantially.

Compositional studies found similarities in protein composition between the pigmented mycelium and the brown mycelial film, as well as between the primordia and the fruit bodies [6]. Therefore, for the isolation of lectins by gel filtration, ion exchange chromatography, and HPLC, three morphological structures were used—nonpigmented mycelium, brown mycelial film, and fruit body. The lectins’ molecular masses and subunit compositions were found by SDS–PAGE.

At the nonpigmented mycelium stage, three highly active lectins were detected, two of which were dimers and the third was composed of four subunits. The subunit molecular masses of the dimeric lectins were 16 and 45 kDa and 16 and 42 kDa. The tetramer’s subunits were of 16, 39, 42, and 45 kDa. The lectins had the highest affinity for l-d-melibiose, d-lactose, and d-galactose.

The lectins of the brown mycelial film contained subunits of 24, 30, and 38 kDa, characteristic only of this morphological structure. By native electrophoresis in polyacrylamide gel, it was shown that these lectins were of about 130, 140, and 150 kDa. Their activity was maximal, as compared to that of the lectins from the other morphogenetic stages. The lectins were specific for the same carbohydrates as were the lectins from the nonpigmented mycelium stage but also for d-galactosamine, *N*-acetyl-d-galactosamine, and *N*-acetyl-d-glucosamine.

The fruit body lectins, whose hemagglutinating activity was lower than that of the vegetative mycelium lectins, were of 43 and 55 kDa. The 43 kDa lectin was more specific for l-d-melibiose and d-lactose and was less specific for *N*-acetyl-d-galactosamine and *N*-acetyl-d-glucosamine. The 55 kDa lectin had an affinity for l-d-melibiose, d-lactose, d-galactose, and d-galactosamine.

The appearance of specific proteins, characteristic only of the mycelial film, is of greatest interest, because it can be related to the transition from one morphogenetic stage to the next. In some basidiomycetes, the appearance of lectins is closely related to the formation of fruit bodies, and it was suggested that lectins can participate in intercellular adhesion [18]. Because the brown mycelial film is a characteristic structure of *L. edodes* that is observed before fruiting and is a tight plexus of thick-walled pigmented hyphae [40], it is logical to assume that its lectins are necessary for hyphal aggregation.

In further work, the development of generative structures was found to be accelerated by the action of a lectin preparation from the *L. edodes* mycelial film on the fungus’s own growing mycelium. After a combined fraction of the brown film lectins was applied to the surface of a 14-day-old mycelium growing on wort agar, the mycelial film took a few days to form and the formation of basidiocarps was two to three times faster than in the control culture. This allowed the assumption that the brown film lectins initiate fruit formation. In addition, it was shown that the *L. edodes* lectins affect the activity of phenol oxidases (laccases) of the fungus in vitro. A nonpigmented mycelium lectin of about 142 kDa and a fruit body lectin of 43 kDa stabilized the activity of laccases isolated from the nonpigmented submerged mycelium, and a brown film lectin of about 150 kDa activated these enzymes [39]. However, the activity of the control laccases, subjected to the same conditions but not incubated with the lectins, decreased almost twofold. In turn, the enzyme-incubated lectins retained their activity at the same level. Probably, the functioning of the enzyme systems, in particular phenol oxidases, can be controlled by lectins, which act as regulators in the process of morphogenesis.

Attempts have been made to find lectins in other *Lentinus* species. Sharma and Atri [41] examined five wild *Lentinus* species (*L. sajor-caju*, *L. connatus*, *L. torulosus*, *L. cladopus,* and *L. squarrosulus* var. *squarrosulus*) for production of an extracellular, loosely surface-bound, intracellular lectin by using hemagglutination with human erythrocytes of the four blood groups (A, B, O, and AB). Among the species tested, only *L. squarrosulus* var. *squarrosulus* expressed lectin activity, with specificity for raffinose, d-sucrose, ribose, and d-maltose. *L. squarrosulus* was also found able to agglutinate erythrocytes of all four human blood groups [42]. A homogenous mycelial lectin of 55 kDa was isolated and purified from *L. squarrosulus* by using a combination of ammonium sulfate precipitation, dialysis, ion exchange chromatography, and gel filtration [9]. The biological activity of this lectin included agglutination of human erythrocytes of all four blood groups and also of goat, sheep, rabbit, and pig erythrocytes. The highest titers were produced with human and rabbit erythrocytes. Neuraminidase treatment of type O erythrocytes considerably augmented the hemagglutination titer. Carbohydrate inhibition studies showed that the lectin had a high affinity for mucin and asialofetuin.

Extracts from the mycelia and/or fruit bodies of a large number of basidiomycetes were screened for the presence of lectins by hemagglutination assays with mouse, rabbit, and sheep red blood cells [10]. Hemagglutinating activity was found in mycelia of *Abortiporus biennis*, *Coriolus* sp., *Ganoderma lucidum*, *Ganoderma resinaceum*, *Grifola frondosa*, *Gymnopilus spectabilis*, *Lentinula edodes*, *Phanaerochaete chrysosporium*, *Pleurotus djamor*, *Pleurotus ostreatus*, *Polyporaceae*, *Punctularia atropurpurascens*, and *Pycnoporus sanguineus*. The carbohydrate specificity of the lectins was determined by hemagglutination inhibition assays with mouse red blood cells by using 43 carbohydrates, including mono-, di-, tri-, and tetrasaccharides, glycoproteins, and polysaccharides. Results for mycelial extracts from *G. lucidum*, *G. resinaceum*, *G. spectabilis*, *P. atropurpurascens*, and *P. sanguineus* showed no clear specificity for a particular monosaccharide tested. The agglutination of *G. spectabilis* was inhibited by galacturonic, glucuronic, and sialic acids. The agglutination of *P. atropurpurascens* mycelial extract was inhibited by the monosaccharides *N*-acetyl-d-glucosamine, glucose, and sorbitol, as well as by several di- and trisaccharides and glycoproteins.

A novel lectin with specificity for *N*-acetyl-d-glucosamine was purified from mycelia of *Punctularia atropurpurascens* by affinity chromatography. The lectin had a subunit molecular mass of 67 kDa and a pI of 5.0. Another lectin was purified from *Pycnoporus sanguineus* mycelium [43]. Its analysis by SDS–PAGE showed the presence of two bands of 68.7 and 55.2 kDa, and the use of ion-exchange chromatography resulted in two bands at pI 5.5 and 5.2. The lectin agglutinated rat erythrocytes and was broadly specific for several monosaccharides, including galactose. Agglutination was also inhibited by the glycoproteins fetal calf fetuin, bovine lactoferrin, bovine transferrin, and horseradish peroxidase.

Several large-scale studies of lectin activity in mycelia and fruit bodies of various basidiomycete species and strains grown by different methods have been made by Davitashvili, Mikiashvili, and their coauthors [7,8,44,45,46,47]. The lectin activity of extracts from the fruit bodies of medicinal and edible mushrooms (13 species), as well as from mycelia grown in liquid culture (6 species, 9 strains) and on malt extract agar (11 species, 23 strains) was evaluated by hemagglutination with rabbit erythrocytes [44]. It was shown that the ability to synthesize lectins is widespread among higher basidiomycetes and that hemagglutinating activity is not only species dependent but also strain dependent. In mycelial extracts, lectin activity was found in *Cerrena unicolor*, *Ganoderma ramnosissimum*, *Ganoderma lucidum*, and *Trametes versicolor.* Comparatively high hemagglutinating activity was present in *C. unicolor* and *G. ramnosissimum* mycelial biomass. Among all fungi tested, *G. ramnosissimum* strain 162 had the highest hemagglutinating activity. Extracts from liquid-cultured mycelia of *C. unicolor* and *T. versicolor* were galactose specific, while those from *G. lucidum* and *G. ramnosissimum* were specific for arabinose and *N*-acetyl-d-glucosamine, respectively.

In another study, 21 strains of higher basidiomycetes belonging to 16 species of different taxonomic and ecological groups were compared for their lectin activity in submerged and solid-state fermentation of agroindustrial wastes or byproducts [45]. Absent or very low hemagglutinating activity was found in *Bjerkandera adusta*, *Lentinus edodes*, *Lenzites varnieri*, *Postia tephroleuca*, *Trametes ochracea*, and *T. versicolor*. By contrast, extremely high specific hemagglutination was detected in the biomass of *Ganoderma applanatum* after solid-state fermentation of lignocellulose. The substrates most appropriate for lectin production were wheat straw (for *Cerrena unicolor*), ethanol production residue (for *Funalia trogii*), and wine bagasse (for *Fomes fomentarius* and *Pseudotremella gibbosa*). Arabinose and mannose were the most frequent inhibitors of hemagglutination.

Comparative examination of *Pleurotus ostreatus* lectins at different growth stages [8] showed that when *P. ostreatus* was grown on wheat straw and a substrate containing distillery effluents, the hemagglutinating activity of mature fruit bodies and vegetative mycelia at harvest reached extremely high values; however, the primordia had very low hemagglutinating activity. Four fractions of proteins (3.5–105 kDa) from the fruit bodies and five fractions of proteins (125, 110, 69, 8, and 1.5–2 kDa) from a vegetative mycelial extract, all with lectin activity, were obtained by gel chromatography. The mycelial lectins were mostly specific for mannose, whereas the highest inhibition toward the fruit body lectins was obtained with *N*-acetyl-d-glucosamine and *N*-acetyl-d-galactosamine for fraction I and with galactose for the other fractions. The heat and pH stability of the lectins and the effect of Ca^2+^, Zn^2+^, Fe^3+^, Cu^2+^, Mg^2+^, and Mn^2+^ on the lectins’ hemagglutinating activity were also investigated.

Two *N*-acetyl-d-galactosamine-specific and one galactose-specific lectin fractions were isolated from the vegetative mycelium of *Cerrena unicolor* HAI 1095 and were characterized [7]. The ability of five strains of two *Cerrena* species (*C. maxima* and *C. unicolor*) to express hemagglutinating activity was evaluated in submerged fermentation of seven lignocellulosic materials of different chemical compositions [46]. Among them, walnut pericarp and leaves were the most appropriate growth substrates for the production of high levels of mycelial lectins. At the same time, absent or very low hemagglutinating activity was recorded in the fermentation of ethanol production residue. Carbohydrate specificity depended largely on both fungal strain and lignocellulosic growth substrate used. The conclusion was made that by substitution of lignocellulosic material, it is possible to regulate lectin production and obtain preparations with different carbohydrate specificities. Proper caution is necessary in evaluating the lectin-producing ability of basidiomycetes.

The ability of *Cerrena unicolor* to elaborate lectins was studied in solid-state fermentation of a mixture of sorghum and wheat straw [47]. The specific hemagglutinating activity toward trypsin-treated rabbit red blood cells in primordial extracts was six-fold lower than that in extracts from mycelial biomass. The hemagglutinating activity of an 80-day-old mycelium increased sixfold in comparison with that of a 55-day-old mycelium. Four protein fractions (160, 105, 67, and 8 kDa) were detected by gel chromatography. Among the sugars tested, galactose was the most potent inhibitor of all protein fractions. The galactose-specific lectins isolated from fractions II and III by affinity chromatography ranged from 15 to 116 kDa and differed in kinetic parameters. The hemagglutinating activity obtained with sorghum and wheat straw as a growth substrate was much higher than that in submerged cultures grown in a glucose-containing synthetic medium and in media containing various lignocellulosic materials. This indicates that a crucial role in lectin accumulation is played not only by the composition of the nutrient medium but also by the method used for fungal growth. Fungi grown under conditions close to natural may be better able to produce proteins than submerged cultures.

A number of studies are devoted to the lectins of plant pathogenic basidiomycetes, such as *Athelia rorfsii* (teleomorph: *Sclerotium rolfsii*) and various *Rhizoctonia* species [48,49,50,51,52,53,54,55,56]. By the using of affinity chromatography, a lectin, RSA (*Rhizoctonia solani* lectin), was isolated from *Rhizoctonia solani* mycelium [48]. It was originally described as a dimeric protein composed of 2 identical subunits of 13 kDa [48], but later, a reinvestigation revealed RSA to be a homodimer of two noncovalently associated monomers of 15.5 kDa [52]. RSA preferentially agglutinated human type A over type B and type O erythrocytes, exhibited specificity towards *N*-acetylgalactosamine, and was also inhibited by several other sugars, including d-fucose but not l-fucose [48]. Localization and sugar specificity of the RSA indicated that this lectin must be different from the earlier discovered l-fucose binding agglutinin associated with the cell wall of *R. solani* [57,58]. It was shown that during the development of *R. solani*, lectin content was low in young mycelium but increased dramatically at the onset of sclerotium formation, reaching a maximum in adult sclerotia [50]. Upon myceliogenic germination, the lectin content of the sclerotia rapidly decreased. The high concentration of the RSA in the sclerotia, and its developmental regulation, indicate that it probably serves as a storage protein in the resting structures of this fungus. Two lectins were isolated from mycelium of *Rhizoctonia crocorum* and *Athelia rolfsii* by affinity chromatography [49]. The *R. crocorum* lectin, RCL (*Rhizoctonia crocorum* lectin), was a tetramer of 11 kDa subunits whereas the *A. rolfsii* lectin, ARL (*Athelia rolfsii* lectin), was a dimer of two 17 kDa subunits. In contrast to the RSA, which exhibited specificity towards *N*-acetylgalactosamine, RCL and ARL had a complex specificity. Another lectin (RBL) has been purified from the mycelium of *Rhizoctonia bataticola*, using ion exchange chromatography and affinity chromatography [56]. This lectin was a tetramer of 11 kDa subunits. The purified RBL was blood group nonspecific and its hemagglutination activity was inhibited by mucin (porcine stomach), fetuin (fetal calf serum) and asialofetuin.

## 3. Lectins on the Surface of Mycelial Hyphae

Most studies devoted to the detection of lectins in the mycelia of basidiomycetes have not considered the location of these proteins in the fungal hyphae. However, it is known that lectins from fungi of different taxonomic groups can be bound to the mycelium surface. This allows lectins to play a role in the recognition process when fungi get involved in symbiotic relationships, parasitic invasion, or nematophagy [57,59,60,61,62].

Elad et al. [57,58] have shown that hemagghitinating substances exist on the surface of plant pathogenic basidiomycete *Rhizoctonia solani* hyphae. The observed significant adherence of red blood cells type O, but not A or B, to *Rhizoctonia* hyphae was inhibited by l-fucose and l-galactose. The lectin present in *R. solani* hyphae also binded to the cell walls of its mycoparasite, *Trichoderma harzianum*, suggesting that the agglutinin may play a role in mycoparasitism process. Agglutination activity was also detected in the soil-borne plant pathogenic fungus *Sclerotium rolfsii* [63]. Culture filtrate and fungal extracts agglutinated certain Gram-negative bacteria and yeasts, but not human erythrocytes. d-Glucose, d-mannose and several of their derivatives specifically inhibited the agglutination of *Escherichia coli* cells. SDS–PAGE of the lectin, purified by gel chromatography, showed two bands with molecular masses of 60 and 55 kDa. The location of the lectin on *S. rolfsii* hyphae and its adsorption to conidia of *Trichoderma* were determined by indirect immunofluorescence [64]. The lectin was adsorbed only to conidia of *T. hamatum* T-244, the antagonist of *S. rolfsii*, and not to the conidia of non-antagonistic *Trichoderma* strains. The adsorption was specifically inhibited by d-glucose or d-mannose. Another lectin was isolated and purified from the culture filtrate of the *S. rolfsii* using a different purification method [60]. SDS–PAGE analysis of the agglutinating fraction revealed a single band corresponding to a protein with a molecular mass of approximately 45 kDa. Agglutination of *E. coli* cells by the purified lectin was inhibited by the glycoproteins mucin and asialomucin, but not by any of the mono- or disaccharides tested. The presence of the purified agglutinin on the surface of the inert nylon fibres specifically induced mycoparasitic behaviour in *Trichoderma harzianum*.

A lectin, LDetL (*Lactarius deterrimus* lectin), was isolated from wild-grown carpophores of the mushroom *Lactarius deterrimus*, a specific symbiont of spruce, by a combination of affinity, hydroxylapatite, and gel-filtration chromatography [11]. Its molecular mass was about 37 kDa, and its structure was dimeric, with two identical subunits assembled by noncovalent bonds. The same lectin was also found in the *L. deterrimus* mycelium, obtained in vitro with young carpophores as the inoculum. Indirect immunofluorescence with antilectin antibodies showed the presence of the lectin in the cell wall. Receptor sites for LDetL were found on the roots, especially on the root hairs, of axenically grown spruce seedlings. Yet, the lectin LDL, previously isolated from the taxonomically related mushroom *Lactarius deliciosus*, a symbiont of pine [32], did not bind to the spruce radicle. This suggests a role for the fungal lectin in recognition and specificity early in mycorrhiza formation.

Oguri et al. [12] found that the basidiomycete *Pleurotus cornucopiae*, strain KC-1, synthesized two lectins, PCL-F and PCL-M, in the developmental stage-specific manner: PCL-F was synthesized in the fruit body, whereas PCL-M was produced only in mycelia grown on a solid medium. The two lectins differed biochemically and immunochemically. PCL-M was isolated from mycelia and was purified to homogeneity by affinity chromatography. Its molecular mass was 40 kDa under reducing conditions, but the subunits were polymerized through disulfide bridges under physiological conditions. *N*-Acetyl-d-galactosamine was the most potent hapten inhibitor. For both *P. cornucopiae* KC-1 and another strain, KC-2, which does not produce PCL-F, no hemagglutinating activity with rabbit erythrocytes was found in extracts of liquid-grown mycelia. PCL-M appeared in mycelia grown on a solid medium before fruit body formation and disappeared during the formation of fruit bodies. It was localized on the surface of solid-grown mycelia and was produced by dikaryotic, but not monokaryotic, mycelia. The authors suggested that PCL-M participates in fruit body formation in *P. cornucopiae* and that it may stimulate the formation of primordia in *P. cornucopiae* by making hyphae adhere to each other. By cloning and sequencing cDNA, the primary structure of PCL-M was determined [65].

Stepanova et al. [66], using native and trypsinized rabbit and human (A, B, AB, and O) erythrocytes, examined hemagglutinating activity in material washed off the surface of agar-grown dikaryotic mycelia of the xylotrophic basidiomycetes *Armillaria mellea*, *Flammulina velutipes*, *Ganoderma applanatum*, *Ganoderma lucidum*, *Grifola frondosa*, *Grifola umbellata*, and *Pleurotus ostreatus*. Agglutinating activity was detected in all cultures, and was stably high throughout the cultivation, the maximum hemagglutination titer was obtained from interaction with native erythrocytes, and only *P. ostreatus* agglutinins had higher titers when human trypsinized AB erythrocytes were used.

From the surface of the dikaryotic mycelium of the basidiomycete *Grifola frondosa*, a lectin was isolated with a molecular mass of 68 ± 1 kDa, consisting of two subunits of 33–34 kDa each [13]. The lectin was a hydrophilic glycoprotein with a protein–glycan ratio of 3:1. Analysis of the amino acid composition of the lectin showed the greatest percentage of amino acids with positively charged R groups, arginine, lysine, and histidine, as well as a complete absence of sulfur-containing amino acids, cysteine, and methionine. d-Glucose and d-glucosamine were detected in the carbohydrate portion. The lectin had a high affinity for native rabbit erythrocytes and human O erythrocytes, but not for trypsin-treated erythrocytes. It had no affinity for any of the tested mono-, di-, and amino sugars or glycoderivatives. Only a polysaccharide, a linear d-rhamnan with the repeating unit structure →2)-β-d-Rha*p*-(1→3)-α-d-Rha*p*-(1→3)-α-d-Rha*p*-(1→2)-α-d-Rha*p*-(1→2)-α-sd-Rha*p*-(1→, blocked hemagglutination completely. Because this configuration of rhamnose had been described only for complex polysaccharides of some microorganisms, Stepanova et al. concluded that the isolated protein was a typical endolectin. This group of lectins recognize only complex oligosaccharides, and agglutination by them can be inhibited only with specific carbohydrate sequences. Conversely, the lectin from the fruit bodies of *G. frondosa*, previously investigated by Kawagishi and coauthors, proved a typical exolectin, because it was highly specific to *N*-acetyl-d-galactosamine [67].

Several immunochemical methods were applied to examine the ability of a lectin from the *G. frondosa* mycelium to interact with homologous and nonhomologous rabbit and human polyclonal antibodies [68]. It was shown by immunodot assay that the lectin bound only to the Fab fragments (antigen-binding center) of homologous antibodies, indicating a specific “antigen–antibody” interaction. The observed interaction of the lectin with nonhomologous antibodies was most likely due to the contact of the carbohydrate-binding region of the lectin with the carbohydrate moiety of the antibodies. Immunofluorescence microscopy with homologous antibodies demonstrated that the lectin was diffusely and unevenly distributed over the surface of the hyphae, forming agglomerates in the region of buckles and young shoots. Lectin complexation with homological antibodies was characterized by greater binding constants, as compared to the use of nonhomologous antibodies [69]. Although lectin affinity to specific and nonspecific antibodies was qualitatively different, essentially the same values of the standard free energy Δ*G*^0^ of these interactions show the universality of carbohydrate–protein interactions at the molecular level.

## 4. Extracellular Lectins

Relatively few studies have searched for and examined extracellular lectins of basidiomycetes, which are released into the growth media during submerged fungal growth. Specifically, Kawagishi et al. [4] detected hemagglutinating activity both in the culture medium and in a crude extract of the *Ganoderma lucidum* mycelium, with the lectins from both sources being identical. On the contrary, a submerged culture of *Laetipous sulphureus* showed the presence of an active lectin in the medium, whereas no lectins were found in the hyphae [14]. In that study, the agglutinating activity of the culture liquid lectin was tested with bromelin-treated human A, B, and O erythrocytes and was found to be low, as compared to the activity of the lectin obtained earlier by the same research team from the fruit bodies of this fungus [70]. It was found that *L. sulphureus*’s extracellular lectin differs from its fruit body lectin.

Stepanova et al. [66] used submerged cultures of ten strains of seven xylotrophic basidiomycete species (*Armillaria mellea*, *Flammulina velutipes*, *Ganoderma applanatum*, *Ganoderma lucidum*, *Grifola frondosa*, *Grifola umbellata*, and *Pleurotus ostreatus*) to estimate the activity of the extracellular hemagglutinins in the culture fluids by using native and trypsinized rabbit erythrocytes and human A, B, AB, and O erythrocytes. When native human and rabbit erythrocytes were used, hemagglutinating activity was observed in the culture liquids of all fungi examined, but during submerged cultivation, it was manifested to a much lower degree, as compared with the activity observed in the material washed off the mycelium during solid-state cultivation of the fungi on agar media. The difference in the taxonomic characteristics and composition of the fungal growth medium in that study exerted far less influence on the activity of the hemagglutinins than the aggregate state of the culture medium and the type of erythrocytes used in the hemagglutination reaction.

Extracellular lectins of *Lentinus edodes* were studied by Tsivileva et al. [15,71]. The hemagglutinating activities of the culture liquid and the submerged mycelium were determined in several *L. edodes* strains [72,73]. The activity of the culture liquids proved much higher than that of the mycelia. The obtained data suggested a considerable amount of extracellular agglutinins being released into the culture medium. Carbohydrate specificity studies demonstrated that the best hemagglutination inhibitors were galactose, lactose, and maltose, which contain galactose and glucose residues. The hemagglutinating activity of *L. edodes* was investigated depending on various cultivation conditions [72,74,75,76,77]. The lectin activity in the culture medium was maximal when the fungus was grown with l-arabinose as a source of carbon and with l-asparagine as a source of nitrogen (C:N ratio, (9.5–12):1) on day 15–18 of culturing at pH 8.0–9.0 [74].

*L. edodes* strain F-249 synthesized two extracellular lectins (L1 and L2) with different characteristics [78]. Both lectins were isolated from the fungal culture liquid and were purified to electrophoretic homogeneity by gel filtration and ion exchange chromatography. The lectins differed in physicochemical properties, composition, carbohydrate specificity, and ability to agglutinate erythrocytes. L1 was a monosubunit glycoprotein of 43 kDa, which contained 10.5 ± 1.0% (*w*/*w*) carbohydrates represented by glucose. L2 was a monosubunit proteoglycan of 37 kDa, which had a carbohydrate content of 90.3 ± 1.0% (*w*/*w*), including glucose (73% of the total mass of the lectin’s carbohydrate moiety) and galactose (27%). The content of asparagine in L2 was high—42% (*w*/*w*) of the total amino acids. This fact, along with the composition of the carbohydrate portion of the molecule (glucose + galactose), allows L2 to be assigned to *N*-asparagine-bound proteins. Both lectins were specific to d-galactose and lactose, with equal minimum inhibitory concentrations of these carbohydrates. Other carbohydrates to which the lectins had an affinity were different for the two lectins: l-rhamnose for L1 and l-arabinose and mannitol for L2. Purified extracellular lectins of *L. edodes* were highly selective in recognizing specific structures on the surface of trypsinized rabbit erythrocytes and did not react with erythrocytes of other animals or of humans.

In a study involving 21 strains of 16 basidiomycete species grown by different methods, several species were found to accumulate proteins with hemagglutinating activity in both culture liquids and mycelial extracts [45]. Moreover, during submerged fermentation of lignocellulose by *Cerrena unicolor* IBB 301 and *Trametes versicolor* IBB 775, the specific hemagglutinating activity was much higher in the culture liquid than it was in the fungal biomass. A more detailed study of extra- and intracellular lectins of a number of *C. unicolor* strains, as well as another species of the genus *Cerrena*, *Cerrena maxima*, involved submerged fermentation of lignocellulosic materials of different chemical compositions [46]. In *C. unicolor* IBB 301, the fermentation of walnut leaves promoted both increase in hemagglutination activity in the biomass and secretion of a large amount of extracellular lectin. Walnut pericarp provided high specific hemagglutination activity in mycelia but was a poor substrate for the formation of extracellular lectin, whereas banana peel stimulated hemagglutination in the culture media but inhibited it in the fungal biomass. In *C. unicolor* IBB 302, only kiwi peel induced an appreciable secretion of hemagglutinins to the culture liquid. Two strains of *C. maxima* had notably different hemagglutination titers, but in both, the culture liquid hemagglutinating activity was highest after submerged fermentation of walnut leaves and pericarp. The specific hemagglutinating activity was low in *C. maxima* culture liquids after fermentation of other lignocellulosic substrates, although many of them favored the formation of large amounts of mycelial lectin.

The hemagglutinating activity of the lectins from the wild (SCW) and mutant (SCM1, SCM2, and SCM3) strains of *Schizophyllum commune* broth was explored by using human blood [16]. The lectins’ activity toward human erythrocytes was found to be more selective to group O, as compared to other blood groups. SCM3 and SCW had the highest and lowest hemagglutinating activities, respectively. An inhibitory study in the presence of sugars demonstrated that the partly purified protein from all *S. commune* strains was a mannose-dependent lectin.

As a summary, Table 1 shows the data for the macrobasidiomycetes whose mycelial lectins have been characterized with respect to their molecular mass and/or specificity.

## 5. Conclusions

In recent years, lectins from basidiomycetes have been the subject of many studies. Interest in these proteins is due to the wide range of their biological activities and to their possible involvement in various aspects of cellular physiology and fungal metabolism.

Most studies reporting on the determination of hemagglutinating activity and the isolation of pure lectin preparations have examined fruit bodies of basidiomycetes. Fruit body lectins have been investigated in several hundred basidiomycete species, including edible, medicinal, and poisonous, and the accumulated data have been reviewed in a number of recent articles [18,19,20,21,22,23]. Less studied are the lectins found in the vegetative mycelium of basidiomycetes. Nevertheless, an analysis of the scientific literature has shown that by now, mycelial lectins have been found in about 40 species of fungi belonging to different taxonomic and ecological groups, and more than half of them are xylotrophs. Pure lectin preparations were obtained only from about a third of these species. These lectins have various biochemical characteristics and carbohydrate specificities. In studies of mycelial lectins, submerged cultivation was primarily used, and solid-state cultivation was used much more rarely. Very few studies have dealt with extracellular basidiomycete lectins, which are found in the growth medium under submerged cultivation conditions. Isolation of lectins from an immersed mycelium and/or from the culture medium is technologically more promising for obtaining mushroom lectins than isolation of lectins from fruit bodies. This method allows full control of the process and, if necessary, the use of regulators for the synthesis of these proteins.

It is important to note that a large number of studies have been devoted to the obtainment of basidiomycete lectins for further use in various fields of biology and medicine [18,19,20,21,22,79]. Yet, there has been insufficient proof of the role of lectins in the metabolism of the fungus itself and in its growth and development. Literature analysis shows that mycelial lectins are no less important than lectins isolated from fruit bodies, and that they deserve more detailed study.

## Figures and Tables

**Table 1:** Characteristics of the lectins obtained from mycelia of macrobasidiomycetes.

Species	Molecular Mass	Sugar Specificity	Reference
Intracellular lectins			
*Agrocybe aegerita*	Homodimeric lectin, 32 kDa	Bovine submaxillary mucin, glycophorin A, hog gastric mucine	[38]
*Armillaria mellea*		Phenyl-β-d-galactopyranoside	[54]
*Cerrena maxima*		Galactose, mannose, lactose	[46]
*Cerrena unicolor*		Galactose	[44]
		*N*-acetyl-d-galactosamine, galactose	[7]
		Mannose, galactose, *N*-acetyl-d-glucosamine	[46]
	15–116 kDa	Galactose	[47]
*Fomes fomentarius*		Arabinose, mannose, fructose, maltose, lactose, raffinose, methyl-α-d-glucose, methyl-α-d-mannose	[45]
*Ganoderma applanatum*		Arabinose, glucose, mannose, galactose, maltose, fructose, *N*-acetyl-d-glucosamine	[45]
		*N*-acetyl-d-galactosamine	[66]
*Ganoderma lucidum*	18 kDa	Asialo-bovine submaxillary mucin, asialofetuin, fetuin	[4]
		Arabinose	[44]
*Ganoderma ramnosissimum*		*N*-acetyl-d-glucosamine	[44]
*Ganoderma* sp.		Arabinose, mannose, fructose, maltose, lactose, methyl-α-d-glucose, methyl-α-d-mannose	[45]
*Grifola umbellata*		Galacturonic acid	[66]
*Gymnopilus spectabilis*		Galacturonic, glucuronic, and sialic acids	[10]
*Lactarius deliciosus*	Dimeric lectin, 37 kDa (subunits of 19 and 18 kDa)	d-Galβ1→3d-GalNAc	[32]
*Lentinus edodes*	Nonpigmented mycelium: two dimeric lectins (subunits of 16 and 45 kDa and 16 and 42 kDa), and one tetrameric lectin (subunits of 16, 39, 42, and 45 kDa)	l-d-melibiose, d-lactose, d-galactose	[6]
	Brown mycelial film: three lectins 130, 140, and 150 kDa	l-d-melibiose, d-lactose, d-galactose, d-galactosamine, *N*-acetyl-d-galactosamine, *N*-acetyl-d-glucosamine	
*Lentinus squarrosulus*		Raffinose, d-sucrose, ribose, d-maltose	[41]
	55 kDa	Mucin, asialofetuin	[9]
*Panafolus papillionaceus*		Glucose, galactose, mannose, fucose, rhamnose, *N*-acetyl glucosamine	[26]
*Pleurotus ostreatus*	Five protein fractions with lectin activity (125, 110, 69, 8, and 1.5–2 kDa)	Mannose	[8]
		*N*,*N*-diacetyl-chitobiose	[66]
*Punctularia atropurpurascens*	67 kDa	*N*-acetyl-d-glucosamine	[10]
*Pycnoporus sanguineus*	Dimeric lectin, 124 kDa (subunits of 68.7 and 55.2 kDa)	Galactose, fetal calf fetuin, bovine lactoferrin, bovine transferrin, horseradish peroxidase	[43]
*Termitomyces clypeatus*		Glucose, galactose, mannose, fucose, rhamnose	[26]
*Trametes versicolor*		Galactose	[44]
*Trametes* sp.		Arabinose, mannose, lactose, raffinose, methyl-α-d-glucose, methyl-α-d-mannose	[45]
*Tricholoma mongolicum*	Two dimeric lectins, both 37 kDa	Lactose	[33]
*Volvariella volvacea*	Homodimeric lectin, 32 kDa	Thyroglobulin	[5]
		Glucose, galactose, mannose, fucose, rhamnose	[26]
Surface-bound lectins			
*Grifola frondosa*	Homodimeric lectin, 68 kDa	Linear d-rhamnan with the repeating unit structure →2)-β-d-Rha*p*-(1→3)-α-d-Rha*p*-(1→3)-α-d-Rha*p*-(1→2)-α-d-Rha*p*-(1→2)-α-sd-Rha*p*-(1→	[13]
*Lactarius deterrimus*	Homodimeric lectin, 37 kDa	β-d-galactosyl(l→3)-d-*N*-acetyl galactosamine	[11]
*Pleurotus cornucopiae*	40 kDa	*N*-acetyl-d-galactosamine	[12]
Extracellular lectins			
*Ganoderma lucidum*	18 kDa	Asialo-bovine submaxillary mucin, asialofetuin, fetuin	[4]
*Lentinus edodes*	Two monomeric lectins, 43 kDa and 37 kDa	L1, galactose, lactose, rhamnose; L2, galactose, lactose, arabinose, mannitol	[78]
*Schizophyllum commune*		Mannose	[16]

## Abbreviations

AAL	*Agrocybe aegerita* lectin
ARL	*Athelia rolfsii* lectin
CGL1	*Coprinus cinereus* lectin 1
CGL2	*Coprinus cinereus* lectin 2
GLL-F	*Ganoderma lucidum* fruit body lectin
GLL-M	*Ganoderma lucidum* mycelial lectin
HPLC	High-performance liquid chromatography
LDetL	*Lactarius deterrimus* lectin
LDL	*Lactarius deliciosus* lectin
SDS–PAGE	Sodium dodecyl sulfate–polyacrylamide gel electrophoresis
PCR	Polymerase chain reaction
RCL	*Rhizoctonia crocorum* lectin
RSA	*Rhizoctonia solani* lectin
TML-1	*Tricholoma mongolicum* lectin 1
TML-2	*Tricholoma mongolicum* lectin 2
VVL	*Volvariella volvacea* lectin
